# Microalgae as Future Foods: Unlocking Their Potential and Overcoming Barriers to Market Adoption and Commercialization

**DOI:** 10.3390/foods15122247

**Published:** 2026-06-22

**Authors:** Tatiele C. do Nascimento, Christian R. Lugcheer, Luisa C. Schetinger, Rafaela Basso Sartori, Mariany Costa Deprá, Adriane T. Schneider, Andressa S. Fernandes, Leila Q. Zepka, Eduardo Jacob-Lopes

**Affiliations:** 1 Bioprocess Intensification Group, Department of Food Technology and Science, Federal University of Santa Maria (UFSM), Santa Maria 97105-900, Brazil; tatielecasagrande@gmail.com (T.C.d.N.); christianlugcheer@gmail.com (C.R.L.); luschetinger@gmail.com (L.C.S.); rafabasso.sartori@gmail.com (R.B.S.); marianydepra@gmail.com (M.C.D.); adriane.schneider@acad.ufsm.br (A.T.S.); zepkaleila@yahoo.com.br (L.Q.Z.); 2 Nutrition and Food Service Research Center, Federal University of São Paulo (UNIFESP), Rua Silva Jardim 136, Santos 11015-020, Brazil; andressa.asfs@gmail.com

**Keywords:** algae, innovative foods, sustainable food sources, food industry challenges, market adoption

## Abstract

For over 70 years, microalgae have been considered promising ingredients for developing sustainable, nutritionally rich foods. Their high protein content, presence of essential amino acids, fatty acids, natural pigments, and a myriad of bioactive compounds position them as potential alternatives to conventional ingredient sources. However, despite their significant potential, the large-scale incorporation of microalgae into food products remains limited. This study presents a critical analysis of the main challenges associated with the use of microalgae in the food industry. Key bottlenecks include high production costs, technological difficulties related to biomass processing, and challenges in extracting desirable compounds. Additionally, the strong flavor, odor, and intense coloration of microalgal biomass can negatively affect sensory acceptance in food products. Other limitations involve scalability issues in cultivation systems, risks of contamination during production, and regulatory constraints related to food safety approval. Consumer perception and limited familiarity with microalgae-based foods also contribute to slower market adoption. Therefore, although microalgae represent a promising and sustainable food resource, overcoming technological, economic, and sensory barriers is essential for their broader integration into the food industry and for achieving successful market consolidation.

## 1. Introduction

The growing global demand for sustainable, nutritionally dense, and functional foods has driven interest in alternative ingredients capable of sustaining the food systems of the future, while reducing the environmental impacts associated with conventional agriculture and livestock production [1]. Current food production models face increasing challenges related to climate change, depletion of natural resources, loss of biodiversity, and increased demand for high-value-added proteins and nutritional compounds [2]. At the same time, greater consumer awareness of the relationship between diet, health, and sustainability has stimulated the development of innovative food ingredients with multifunctional properties and lower environmental impact [3]. In this context, microalgae stand out as one of the most promising biotechnological platforms for the development of next-generation foods.

For more than seven decades, microalgae have been explored as potential food ingredients due to their high productivity and wide biochemical diversity. These microorganisms are capable of synthesizing high concentrations of proteins, polyunsaturated fatty acids, pigments, polysaccharides, vitamins, minerals, and various bioactive compounds of nutritional and functional interest [4]. In addition to their nutritional value, they exhibit important techno-functional properties, including emulsification, foaming, gelation, water retention, and thickening, as well as biological activities, especially antioxidants, expanding their applicability in food formulations [5]. Despite the extensive microalgal biodiversity, only a few species have achieved commercial relevance for biomass production, such as *Arthrospira* and *Chlorella*, or for obtaining high-value-added compounds, including astaxanthin from *Haematococcus pluvialis*, β-carotene from *Dunaliella salina*, and the fatty acids DHA and EPA produced by *Schizochytrium* and *Nannochloropsis* [6]. These examples reinforce the high industrial and economic potential of these microorganisms.

However, despite the benefits associated with microalgae, their effective incorporation into large-scale food systems remains limited. The transition from experimental potential to industrial consolidation is hampered by interconnected barriers related to cultivation, biomass processing, scalability, sensory acceptance, and regulatory approval [7]. High production costs, low industrial standardization, contamination risks, high energy consumption during processing, and the compositional variability of biomass compromise economic competitiveness compared to conventional agricultural ingredients [8,9]. Furthermore, intense sensory characteristics, such as color, odor, and characteristic flavor, often reduce consumer acceptance, requiring technological strategies focused on sensory optimization and product reformulation [5]. In addition to this, there are regulatory challenges, since legal frameworks remain heterogeneous across different countries, hindering the commercialization and international expansion of these products [10].

Although several emerging technologies have been proposed to increase the sustainability and efficiency of microalgal processing, many remain restricted to laboratory or pilot scales, exhibiting limited industrial maturity [9]. Furthermore, despite the multifunctionality of microalgal compounds representing a significant advantage, the high compositional variability among species, cultivation conditions, and processing methods still compromises predictability and functional reproducibility in complex food matrices [11,12].

Despite the growing recognition of microalgae as promising resources for the sustainable production of functional foods, significant scientific, technological, industrial, and regulatory barriers still hinder their large-scale adoption. In this context, the present review provides a critical and integrated assessment of the nutritional composition, techno-functional properties, biomass processing strategies, industrial scalability, economic feasibility, regulatory frameworks, sensory acceptance, and market dynamics associated with microalgae-based foods.

While previous reviews have primarily focused on specific topics, such as nutritional composition, protein production and extraction, techno-functional properties, sustainable food development, or regulatory aspects, these dimensions are often addressed separately. In contrast, this review adopts a translational perspective, examining how these factors interact throughout the pathway from biomass production to market adoption. By integrating these interconnected dimensions, the review seeks to bridge the gap between the recognized potential of microalgae and the practical challenges that continue to limit their commercial deployment. Furthermore, it highlights the emerging role of microalgae as sources of high-value functional ingredients and bioactive compounds, while discussing the key opportunities and challenges that will shape their future incorporation into global food systems.

## 2. Literature Search Strategy

This review was conducted with the aim of integrating current knowledge on the use of microalgae as food ingredients, with emphasis on nutritional properties, technofunctional characteristics, biomass processing, industrial scalability, economic viability, regulatory aspects, and market adoption. The analysis sought to provide a comprehensive overview of the advances, challenges, and perspectives related to the incorporation of these biomasses into food systems, contributing to the understanding of the factors that influence their consolidation in the food market. The bibliographic search was carried out using the Scopus, Web of Science, PubMed, and Google Scholar databases. The main search terms included combinations of the keywords “microalgae”, “food applications”, “foods of the future”, “proteins”, “lipids”, “polysaccharides”, “pigments”, “bioactive compounds”, “food ingredients”, “technofunctional properties”, “biomass processing”, “sustainable food systems”, “economic viability”, “industrial scalability”, “food regulation”, “consumer acceptance”, and “commercialization”. The searches mainly covered publications between 2010 and 2026, although seminal studies published outside this period were also included when considered essential for contextualization. Articles were selected based on their relevance to the review’s objectives, prioritizing original peer-reviewed research articles, review articles, book chapters, regulatory reports, and techno-economic assessments. Studies focused exclusively on biofuel production, wastewater treatment, or non-food applications were generally excluded unless they provided information directly relevant to food production, processing, or marketing. The selected literature was critically evaluated and synthesized to identify the main opportunities, challenges, and knowledge gaps associated with the large-scale adoption of microalgae in food systems.

## 3. Caloric and Nutritional Value

Microalgae generally exhibit high nutritional density, characterized by a high concentration of macronutrients—especially proteins, lipids, and carbohydrates—as well as micronutrients such as minerals and vitamins, and various bioactive compounds relevant to metabolism [4]. Among the commercially established and widely distributed species, *A. platensis* (commercially known as Spirulina), *Chlorella vulgari*, *Haematococcus pluviali*, and *Dunaliella salina* stand out [13]. In this context, Table 1 presents a nutritional and energy comparison of these microalgae in relation to conventional foods, based on data from the literature and regulatory and informational databases.

According to the USDA database [14], Spirulina (290 kcal/100 g) and *Chlorella* (300–400 kcal/100 g) generally have a higher energy density when compared to widely consumed foods worldwide, such as beef (260 kcal/100 g), chicken (106–119 kcal/100 g), eggs (146–150 kcal/100 g), potatoes (52–77 kcal/100 g), and rice (129 kcal/100 g). This high energy value is related to the chemical composition of the biomass, especially the levels of protein (56–60 g/100 g), carbohydrates (20–40 g/100 g), lipids (0–10 g/100 g), and ash (6 g/100 g). Comparatively, Spirulina biomass stands out for its high ash content, reflecting its high mineral concentration, while other foods generally have values lower than 1 g/100 g.

### 3.1. Protein Fraction

Species such as *A. platensis* (60–70%) and *Chlorella vulgaris* (42–60%) stand out for their high protein content; however, this content can exceed 70%, depending on the genus, species and cultivation conditions [24].The nutritional quality of proteins is mainly determined by the essential amino acid profile, directly related to the biological value or chemical score. The greater the adequacy of this profile to human requirements, the greater its metabolic efficiency tends to be after ingestion. From this perspective, microalgal proteins have often been described as comparable to conventional protein sources [25,26]. More detailed information on the essential amino acid profile can be found in [27,28].

In this context, amino acids from microalgae can complement those that are limiting in conventional proteins. Beef, pork, and chicken tend to have lower levels of tryptophan and methionine, while milk and dairy products are relatively deficient in sulfur-containing amino acids, such as methionine and cysteine [29]. In parallel, strategies for optimizing cultivation conditions have been investigated with the aim of increasing the protein content and, consequently, the production of microalgal amino acids [30]. The high nutritional value combined with the potential for sustainable production has consolidated microalgal biomass as a promising food source for the future [31], especially Spirulina, often described as a “superfood” due to its high nutritional density and high concentration of bioactive compounds [32].

Despite their promising nutritional profile, the widespread adoption of microalgae as food sources remains constrained by the structural complexity of their cell walls. In numerous species, the highly resistant cell wall acts as a physical barrier that hinders the release and digestion of intracellular components, ultimately compromising protein digestibility and the bioavailability of key nutrients [33]. For this reason, strategies aimed at disrupting the cell wall have been investigated as alternatives to increase the bioaccessibility and metabolic utilization of nutritional compounds [34]. In the absence of these approaches, the high protein content and the presence of essential amino acids do not always translate into effective nutritional utilization by the body.

In summary, the nutritional value of proteins should not be attributed exclusively to high protein content or the presence of essential amino acids. Parameters such as digestibility, bioavailability, and antinutritional factors exert a decisive influence on the metabolic efficiency of these compounds after ingestion [35]. Although certain microalgal species can be effectively valued as promising protein sources, the extrapolation of this potential to the entire microalgae population remains limited. A large part of these microorganisms continues to be restricted to the field of research and development, often associated with perspectives aligned with global demands for more sustainable and healthy foods, which still require technological, nutritional, and economic validation for viability on an industrial scale.

### 3.2. Nonpolar and Polar Lipid Fraction

Microalgal lipids constitute an important fraction of biomass and contribute significantly to its nutritional and functional value. Several species, including *Chlorella* spp., *Isochrysis galbana*, *Nannochloropsis oculata*, *Pavlova lutheri*, *Schizochytrium* sp., and *Auxenochlorella protothecoides*, are recognized for their lipid-producing capacity, with reported lipid contents of approximately 16%, 24%, 19%, 25%, 32%, and up to 70% of dry biomass, respectively [36]. Lipid accumulation is strongly influenced by both species-specific characteristics and cultivation conditions and can be enhanced through environmental and nutritional strategies designed to induce lipid biosynthesis. Under optimized conditions, certain oleaginous microalgal strains may achieve lipid productivities that rival or exceed those of conventional oilseed crops [37]. This fraction of microalgae is composed predominantly of polar lipids and, to a lesser extent, of non-polar lipids [38].

Among the non-polar lipid compounds produced, long-chain polyunsaturated fatty acids (PUFAs) stand out, especially eicosapentaenoic acid (EPA) and docosahexaenoic acid (DHA), widely recognized for their associated benefits to cardiovascular, neurological, and immunological health [4]. Due to this biosynthetic capacity, oils obtained from *Schizochytrium* sp. and *Nannochloropsis* sp. are currently produced on an industrial scale and marketed globally as important sources of DHA and EPA, respectively [6]. In this context, microalgae emerge as a sustainable alternative for obtaining these essential lipids, traditionally derived from marine organisms, whose intensive exploitation has raised increasing environmental and sustainability concerns [39].

Recently, the fraction of polar microalgal lipids has attracted increasing attention due to its high in vivo mobility, favoring intestinal absorption and the transport of bioactive compounds to the bloodstream and target tissues. Unlike triglycerides (the conventional delivery system), these molecules exhibit greater stability and bioavailability, characteristics that can enhance the physiological utilization of polyunsaturated fatty acids, especially those of the omega-3 series. Among polar lipids, phospholipids and glycolipids stand out not only for acting as efficient carriers of EPA and DHA, but also for exhibiting their own biological activities, including anti-inflammatory effects [40]. A recent study with polar lipid fractions obtained from *Nannochloropsis oceanica* and *Chlorococcum amblystomatis* demonstrated a significant reduction in nitric oxide production and the expression of pro-inflammatory genes in macrophages stimulated by lipopolysaccharide. The most significant effects were observed in the fractions enriched in diacylglyceryl-trimethylhomoserine and phospholipids of *N. oceanica* and in digalactosyldiacylglycerol and sulfoquinovosyldiacylglycerol of *C. amblystomatis* [41].

Despite the important role played by polar lipids in microalgae, knowledge about their structural diversity and potential biological effects remains limited when compared to other classes of bioactive compounds. In this context, using LC-MS analyses, Conde et al. [42] identified approximately 150 species of oxidized polar lipids distributed among different classes of phospholipids, glycolipids, and betaine lipids in five microalgae species (*Chlorella vulgaris*, *Chlorococcum amblystomatis*, *Scendesmus obliquus*, *Nanochloropsis oceanica*, and *Phaeodactylum ticornutum*). These results highlight the high complexity of this microalgal lipid fraction and reinforce its potential as a source of bioactive molecules that are still poorly explored.

### 3.3. Carbohydrate Fraction

Microalgal carbohydrates, mainly composed of different polysaccharides, can account for more than 60% of the biomass, depending on the species and cultivation conditions [27]. These biopolymers perform structural and energy storage functions in cells, and some of them can also be excreted under normal physiological conditions or in response to environmental stress [43]. In addition to their metabolic importance, the polysaccharides produced by microalgae have relevant technofunctional properties, standing out for their potential application as stabilizing and texturizing agents in the food industry [44]. Among the species of greatest biotechnological interest for the production of these compounds, *Porphyridium cruentum* stands out for its high potential for polysaccharide biosynthesis, mainly exopolysaccharide [45].

The exopolysaccharides excreted by microalgae exhibit high structural complexity, containing different monosaccharides and non-sugar substituents, especially sulfate groups, characteristics that differentiate them from conventional polysaccharides and are directly associated with their bioactivity [46].

Several studies have associated these compounds with anticoagulant, immunomodulatory, antitumor, hypoglycemic, anti-inflammatory, and antioxidant activities. However, the great chemical and structural diversity of these biopolymers makes it difficult to establish clear relationships between structure and function, limiting the understanding of their mechanisms of action, the characterization of their properties, and the exploration of their potential in industrial applications [47].

### 3.4. Micronutrients and Bioactive Compounds

Regarding micronutrients, microalgae are capable of synthesizing a wide range of vitamins with significant biotechnological potential, notably β-carotenes, precursors of vitamin A, as well as vitamins B1, B2, B6, B12, C, and E [48]. The mineral content of these microorganisms generally represents between 2.2% and 4.8% of the dry biomass, encompassing elements such as potassium, iron, magnesium, zinc, sulfur, copper, phosphorus, and iodine [49,50].

In addition, microalgae represent an important source of bioactive pigments, including carotenoids, phycocyanins, phycoerythrins, and chlorophylls, compounds that, in addition to contributing to the coloration of foods, have relevant functional properties [4,36,51,52]. Among the species with the greatest potential for carotenoid production, *Dunaliella salina* and *Haematococcus pluvialis* stand out commercially due to their high capacity for biosynthesis of these pigments [53]. Phycobiliproteins, in turn, are mainly produced from genera such as Spirulina, *Porphyridium*, and *Rhodella*, with phycocyanin being particularly relevant as it constitutes one of the main natural blue dyes available for industrial application [54,55]. In contrast, the biotechnological exploration of microalgal chlorophylls is still relatively recent. These pigments are responsible for the characteristic green coloration of chlorophytes and cyanobacteria and have been arousing increasing interest due to their potential as a functional and bioactive ingredient [56,57].

Due to this diverse chemical composition, microalgae have high nutritional and functional potential, contributing to the development of enriched food formulations and bioactive ingredients.

## 4. Techno-Functional Properties

The generation of compounds with high nutritional density, combined with the ability to perform technological, sensory, and bioactive functions, supports microalgae entry into emerging markets [7,58]. The multifunctionality attributed to microalgae reflects their diversity of biocompounds. However, this diversity of compounds, presented as an advantage, also brings functional variation and technological limitations in functional application, industrial standardization, and stability throughout processing, especially when applied to heterogeneous food matrices [11,12]. Among its techno-functional properties, the following stand out: antioxidant capacity, thickening, emulsification, foam formation, and gelation.

The sensory properties of microalgae highlight their dual role in providing techno-functional benefits while simultaneously contributing to health-promoting effects. However, this duality is often antagonistic, as many of the compounds responsible for physiological benefits are also associated with undesirable sensory characteristics. The remarkable diversity of chemical structures present in microalgal biomass can modulate sensory perception, including taste and aroma attributes, thereby directly influencing consumer acceptance [5,59].

Thus, compounds such as polyphenols, terpenoids, organosulfur compounds, alkaloids, peptides, and amino acid derivatives are presented as multifunctional components present in microalgae capable of modulating sweetness, bitterness, umami, kokumi, spiciness, astringency, and characteristic aromatic profiles [60,61]. Therefore, food reformulation strategies based on these characteristics offer the possibility of minimizing the use of sodium, sugar, and fat in new formulations. However, these approaches often result in complex technologies and processing costs, and they do not eliminate consumer opposition, one of the main bottlenecks that persist for large-scale adoption [62,63]. Although this resistance is justified by the association with earthy, marine, or intensely herbaceous notes, technological interventions are necessary to mitigate it in order to design next-generation products that promote health without compromising flavor.

In light of this, the techno-functional properties of the components present in microalgae play a decisive role in food formulations. These techno-functional properties govern the behavior of these compounds during preparation, processing, and storage, influencing the texture, stability, and overall performance of the product [59]. Table 2 presents some of the techno-functional properties offered by microalgae biomass for application in food.

In parallel, due to the presence of bioactive compounds (carotenoids, chlorophylls, phycobiliproteins) in their composition, whole microalgae biomass can contribute to the inhibition of lipid oxidation through mechanisms of electron donation, hydrogen transfer, and chelation of pro-oxidant metals [65]. However, trade-offs between technological functionality and sensory impact resulting from intense color and chemical reactivity still require further development for the application of these compounds. Thus, the use of microalgae in food matrices offers an emerging alternative with multiple functions. In addition to their potential as natural colorants and additives, pigments perform bioactive functions. For example, *Chlorella* biomass exhibits antioxidants, antidiabetic, immunomodulatory, antihypertensive, and antihyperlipidemic effects in humans and animals. Phycocyanin from *Arthrospira* demonstrates anticancer properties, controlling the growth of tumor cells. Furthermore, fucoxanthin from marine microalgae has anti-obesity and anti-cancer benefits [66].

Conversely, although its application is positive in food products, process requirements are presented. Despite the importance of its antioxidant activity, the same chemical structure is also responsible for imparting an intense color, restricting its application in a portion of food formulations. Also, when exposed to light, chlorophylls can act as pro-oxidants and form singlet oxygen, which accelerates oxidative processes. Singlet oxygen is produced during the transfer of energy from excited chlorophyll to molecular oxygen in the process of photosynthesis [67].

Concerning structural properties, compounds such as functional polysaccharides, proteins and protein-derived fractions, lipophilic bioactives, and antioxidant-rich extracts exhibit the formation of three-dimensional networks resulting from intermolecular interactions, including hydrogen bonds, hydrophobic interactions, and electrostatic associations [68,69]. However, predicting how these interactions occur in food matrix formulations, which are mostly complex, is still limited, mainly due to the compositional variability of microalgae biomass. Given this premise, microalgae-based compounds can even be used as fat substitutes in food formulations intended for specific audiences [58,63].

It is important to emphasize, however, that the structural microalgae properties vary according to the molecular mass of the proteins. The microalga Chlorella, for example, can provide proteins with a molecular mass of 14–23 kDa, which tend to exhibit greater solubility and diffusion, favoring emulsifying and foaming properties. Proteins with higher molecular weight, from 28 to 116 kDa, have greater viscosity and gelling potential. Furthermore, the presence of essential amino acids (histidine, isoleucine, leucine, lysine, methionine, phenylalanine, threonine, and valine) increases nutritional value and contributes to conformational stability and intermolecular interaction capacity, providing better thermal stability and technological functionality in food matrices [70,71]. In addition to essential amino acids, microalgae also synthesize non-essential amino acids, most notably glutamic acid, which is associated with umami taste and can enhance the perception of salty flavor in food preparations. Therefore, these compounds can be explored as a nutritional strategy to reduce sodium consumption, currently recognized as a major global public health problem.

The insertion of *A. platensis* into a soy protein isolate hydrogel corroborated the gel’s increased strength, hardness, and storage modulus. These characteristics were attributed to the dissolution of polysaccharides present in the cell wall of the three-dimensional network of isolated soy protein hydrogel, resulting in greater physical interactions and a higher number of β-sheets in the protein structure [72].

The interfacial properties of microalgae with emulsifying and foaming action are mainly associated with their lipid and protein fractions, respectively. For an emulsion or foam to form between two immiscible fluids—water and oil in the case of emulsions, and water and air in the case of foams—there must be an area of interfacial contact with unfavorable energy caused by the interfacial tension between the two phases [73]. Although the emulsifying capacity of these microalgae-derived compounds makes them a viable alternative to conventional proteins, their practical application requires improvements related to their lower conformational flexibility and reduced solubility under industrial conditions. However, when the bioactive lipid compounds from microalgae are incorporated into food matrices, they provide attributes such as texture, juiciness, and stability, especially in plant-based products and meat analogues. Furthermore, processing conditions such as pH, ionic strength, and pretreatment of biomass impose restrictions on its application in standardized formulations.

Moreover, extrinsic conditions related to processing and intrinsic conditions of microalgal biomass govern the ideal formation of the emulsion [30]. For example, the microalgal lipid fraction has high concentrations of lipophilic surfactants that act by reducing interfacial viscosity. Thus, these surfactants can help stabilize inverse O/W emulsions. Studies suggest that lipids derived from microalgae crystallize at the interface, allowing for the stabilization of oil-air interfaces and fat foams [73].

Regarding foam-forming properties, microalgae-based protein compounds provide the ability to adsorb at water-air interfaces, minimizing interfacial tension and promoting physical stability in dispersed systems. Although microalgae proteins exhibit lower solubility and flexibility characteristics than conventional base proteins, emerging technology strategies such as enzymatic hydrolysis and physical modification can be employed to improve these limitations during food applications [12,74].

Despite the various technofunctional properties presented, there are significant limitations in their applications. Among these, the following stand out: low solubility, chemical instability, and undesirable flavor profiles between batches of biomass, which compromise reproducibility on a large scale, as well as limited bioavailability and reduced bioactivity in real-world systems when applied to food matrices [7,75].

To overcome these bottlenecks, robust nano- and microencapsulation approaches have been proposed as promising technological strategies to expand the application of these compounds in food as a functional ingredient. Encapsulation involves trapping or coating an active agent within a carrier material, resulting in a particulate system capable of accommodating substances in solid, liquid, or gaseous forms. By way of example, studies show that encapsulation of astaxanthin resulted in greater stability during processing and superior bioavailability compared to the free form [76]. Furthermore, various encapsulation methods can be employed, such as lipid carriers, nanocapsules loaded with lutein, liposomes, or sodium alginate, each favoring the benefits of greater bioavailability of the biocompounds [7]. Nevertheless, limitations in scalability and operational costs of these technologies remain significant barriers to effective implementation in large-scale industries.

Importantly, simply optimizing bioavailability does not necessarily imply maximizing biological functionality. Thus, the concept of bioactive synergy emerges as an advanced level of functional integration, seeking to simultaneously enhance the technological and physiological effects of two or more compounds when combined. Despite its relevance, practical application remains challenging due to complex interactions among compounds within real food matrices during the development of functional formulations. Indeed, interactions between natural compounds can further amplify the positive health effects. For the application of microalgae in complex food matrices, this concept is particularly relevant due to the multiple biochemical and physicochemical mechanisms that occur simultaneously in these formulations [77,78].

In lipid-based systems, for example, the bioavailability of carotenoids is influenced by dietary lipids, which enhance their micellar solubilization and intestinal absorption, thereby improving their functional efficacy [79]. Regarding other antioxidants, the bioactive synergy enhances the ability to neutralize reactive compounds, demonstrating high oxidative stability when applied to food matrices [78]. However, such interactions are highly dependent on the composition of the food matrix and the processing conditions employed, which limits the predictability of their effects in real-world applications.

Based on the above, understanding the nature and behavior of bioactive compounds becomes a fundamental step in advancing the application of these interactions. Thus, knowledge of the main bioactive compounds present in microalgae with the potential to act in functional synergies, as well as the mechanisms involved in these interactions, can support the development of robust research strategies aimed at formulating innovative foods.

The connection between sensory, technological, and bioactive aspects positions microalgal compounds as strategic elements for the development of innovative food systems. These compounds can provide techno-functional properties, such as emulsifying, foaming, gelling, and thickening capacities, in addition to health benefits comparable to those associated with conventional ingredients currently available on the market. However, the effective advancement of microalgae applications in the food sector and their market consolidation still depend on overcoming structural bottlenecks, including biomass standardization, functional predictability, process scalability, and consumer acceptance. In this context, optimizing biomass production and processing steps represents a fundamental strategy to enable their industrial application.

## 5. Biomass Processing

**Figure 1:**
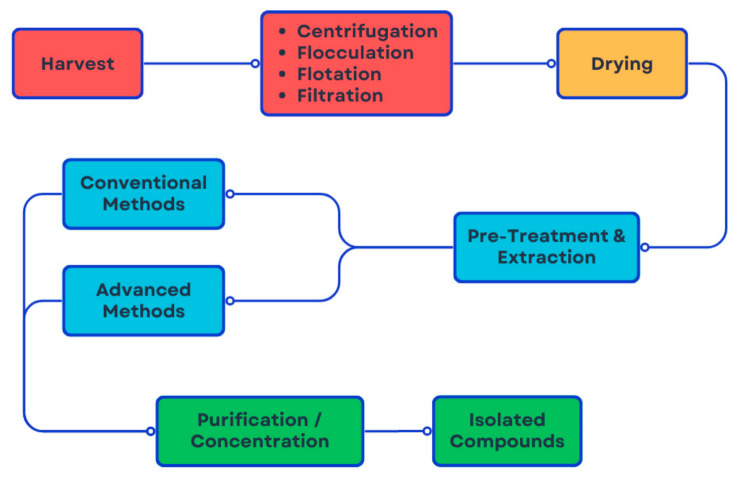
General flow of microalgal biomass processing.

Microalgal biomass processing is essential for its industrial application and encompasses both conventional steps, such as harvesting, drying, and extraction, and emerging technologies aimed at process simplification, efficiency, and cost reduction. The selection of these techniques should consider not only metabolite recovery efficiency, but also factors such as energy consumption, scalability, and preservation of bioactive compounds. In this context, Figure 1 summarizes the main biomass processing routes, including harvesting, drying, and extraction, highlighting their advances, limitations, and future perspectives.

After defining the optimal harvesting point (generally corresponding to the maximum biomass productivity or yield of the target compound), the separation of the biomass from the culture medium is initiated. Among the most commonly employed harvesting methods are filtration, centrifugation, flocculation, and flotation [13].

Filtration is particularly suitable for more fragile microalgal species and consists of separating the biomass through semipermeable membranes, with the filtration system varying according to the characteristics of the microalga and the culture medium. In contrast, centrifugation promotes the physical separation of biomass from the liquid medium through centrifugal force, enabling the recovery of approximately 80–90% of the biomass within a short period and without the need for chemical additives, although high centrifugal forces may cause structural damage to the cells. Flocculation is based on the aggregation of microalgal cells into larger particles through the addition of flocculating agents, which may be biological, such as bacteria, fungi, and biomolecules, favoring a more sustainable harvesting process, or chemical, such as iron and aluminum salts, whose use may generate toxic by-products and increase environmental risks. Finally, flotation represents an economically viable alternative for large-scale processes, employing microbubbles that adhere to microalgal cells and promote their migration to the culture surface, forming a concentrated biomass-rich foam [13].

The subsequent biomass processing step corresponds to drying, whose primary purpose is to reduce water activity, thereby improving microbiological stability and minimizing degradative reactions during storage. However, this operation presents important limitations, since exposure to heat may compromise the stability of sensitive compounds. In this context, conventional drying systems, such as bed and tray dryers, are widely employed, although they require carefully controlled operating conditions to minimize thermal damage to target compounds [80].

Spray drying also presents relevant drawbacks, mainly due to the exposure of biomass to elevated temperatures capable of negatively affecting the stability of thermosensitive bioactive compounds. Although the short residence time may partially mitigate these effects, degradation remains a significant concern, particularly for pigments and other heat-sensitive metabolites. In addition, this technique is associated with high energy consumption, which may compromise its economic feasibility for certain industrial applications [81].

Conversely, freeze-drying, widely applied at laboratory and pilot scales, stands out for its high efficiency in preserving the structural and bioactive properties of biomass, as it prevents thermal degradation of compounds. Nevertheless, its industrial application remains limited due to high operational costs associated with specialized equipment operating under freezing and vacuum conditions, which require substantial energy input and skilled labor [82].

As an alternative, solar drying has been investigated as a more sustainable and economically attractive approach for the large-scale production of Tetraselmis chui and Nannochloropsis oceanica biomass [83]. This study demonstrated that the technique is capable of preserving the overall chemical composition of biomass, including proteins, carbohydrates, lipids, and fatty acid profiles, similarly to freeze-drying. However, direct exposure to solar radiation resulted in significant reductions in carotenoid and chlorophyll contents. Therefore, despite its economic and environmental advantages, solar drying still shares limitations commonly associated with thermal methods, particularly regarding the preservation of photosensitive compounds.

Regarding extraction methodologies, these are generally integrated with pretreatments aimed at cell disruption and are frequently performed immediately after biomass drying. Cell disintegration is essential to promote the release of intracellular compounds, since microalgae possess highly resistant cell walls developed for protection and transport functions, mainly composed of complex polysaccharides [84]. For this purpose, several conventional and emerging strategies have been employed. Among the most widely used techniques are bead milling, solvent extraction, maceration, osmotic shock, as well as ultrasound- and microwave-assisted methods [85].

Among these techniques, bead milling consists of a mechanical cell disruption method employing grinding and dispersion equipment fitted with a central rotating shaft that promotes collisions between microalgal cells and glass or ceramic beads, resulting in cell wall rupture. Process efficiency is directly related to factors such as bead concentration and diameter, as well as the structural characteristics of the treated cells [86].

Solvent extraction is based on the interaction between microalgal biomass and a solvent presenting chemical affinity for the target compounds (generally, acetone, acetic acid, chloroform, dichloromethane, diethyl ether, ethanol, and hexane.) This interaction facilitates solvent diffusion into the cells, metabolite solubilization, and subsequent migration of the solvent–compound complex to the external medium, enabling compound recovery [87].

This process may be combined with maceration, a technique involving biomass size reduction followed by immersion in organic solvents, with or without heating, in order to enhance mass transfer and intracellular compound diffusion. After extraction, the solvent is typically subjected to centrifugation, filtration, and evaporation steps for extract concentration. Despite its operational simplicity and broad laboratory-scale application, this methodology presents limitations associated with high solvent consumption and prolonged extraction time, factors that increase the environmental impact of the process [88].

In contrast, osmotic shock is characterized as a physicochemical pretreatment based on the application of osmotic gradients capable of increasing intracellular pressure and promoting cell wall rupture. This strategy has been mainly applied for the recovery of lipids, proteins, and carbohydrates and may also be combined with ultrasound and enzymatic treatments to improve extraction efficiency, especially for proteins [89,90].

Microwave irradiation promotes cell disruption through the combined action of electromagnetic fields and thermal heating resulting from the interaction of microwaves with polar and dielectric molecules, particularly water present in the biomass. This process generates rapid internal heating, favoring cell wall disintegration. In contrast, ultrasonication is based on the propagation of sound waves capable of generating successive compression and decompression cycles, leading to microbubble formation in the medium. The collapse of these bubbles produces localized high pressures, heat, free radicals, and shock waves, promoting cellular disruption and the release of intracellular compounds [89,91]. Both techniques are considered more advanced approaches among conventionally employed methods for microalgal compound extraction and present the advantage of reducing the use of highly toxic solvents. However, microwave application may favor the degradation of thermosensitive compounds, whereas ultrasound still faces challenges related to industrial-scale implementation.

Overall, these methodologies still present important limitations, including high environmental impact associated with organic solvent use, low selectivity, difficulties in industrial scale-up, and potential degradation of extracted compounds under severe processing conditions. Consequently, interest in more sustainable and environmentally friendly emerging technologies has increased, particularly high-pressure processing, pulsed electric fields, ohmic heating, ionic liquids, and deep eutectic solvents [85].

Among these approaches, high-pressure homogenization is a cell disruption technique that employs a high-pressure pump. During processing, cell suspensions are subjected to elevated pressures while passing through micrometric valves, exposing cells to intense mechanical stress caused by turbulence, shear, elongation, and cavitation, ultimately promoting cell rupture. Its main advantages include low energy consumption, scalability, and continuous processing capability [92].

Pulsed electric field (PEF) technology involves the application of intense, short-duration electric pulses to the microalgal suspension, increasing cell membrane permeability through electroporation. This process intensifies intra- and extracellular exchange, favoring compound extraction, particularly lipids. Due to its efficiency, PEF has attracted considerable attention as an innovative technology for industrial food processing and the extraction of microalgal biomolecules, especially pigments [85,93].

While pulsed electric fields are widely recognized for predominantly inducing non-thermal effects, ohmic heating corresponds to the thermal effect generated by the application of a moderate electric field. The intensity of this thermal effect depends on both the applied electric field and sample composition. Heating occurs due to heat generation during the passage of electric current through a semiconductive matrix, such as microalgal biomass, and is proportional to the intrinsic electrical resistance of the material, according to Joule’s law. Thus, the rapid conversion of electrical energy into thermal energy constitutes an important advantage of this technique, as it promotes accelerated membrane disruption and intracellular compound solubilization while reducing prolonged exposure of bioactive compounds to high temperatures compared with conventional heating methods [94]. This technology has been applied for the extraction of microalgal chlorophylls and carotenoids [85,95].

Ionic liquids are composed of a cation and an anion responsible for their physical and chemical properties, respectively. Their properties can be tailored through structural modifications, enabling the customization of physicochemical characteristics according to the intended application [96]. In this context, these solvents have shown promising results for lipid extraction from wet microalgal biomass, allowing processing at lower temperatures and shorter times compared with conventional extraction methods [97]. Furthermore, recent studies have demonstrated their potential for pigment extraction from dried biomass when combined with ultrasound [98].

Recently, deep eutectic solvents have gained increasing attention as sustainable alternatives to conventional solvents due to their ability to enhance mass transfer and improve solvent penetration into biomass. These systems are formed by combining hydrogen bond acceptors, such as choline chloride, choline acetate, betaine, and menthol, with hydrogen bond donors, including organic acids, sugars, amides, amines, and alcohols [99]. Combinations such as choline chloride:acetic acid and choline chloride:urea have been employed for the extraction of proteins, carotenoids, phenolic compounds, carbohydrates, and lipids from microalgae, frequently associated with ultrasound application to enhance cell disruption and intracellular compound release [100].

Considering the challenges identified, an alternative strategy to mitigate the limitations associated with biomass processing involves the direct utilization of whole biomass, either wet or dried, thereby avoiding costly drying and/or extraction steps. However, this approach still presents important limitations. In the case of wet biomass, the main restriction is the need to maintain a cold chain in order to preserve physicochemical, microbiological, and bioactive properties. In addition, for functional applications, both wet and dried biomass require cell disruption treatments to increase bioavailability and optimize the biological utilization of bioactive compounds [101]. Conversely, when the goal is the extraction of isolated compounds, the biorefinery approach can be economically advantageous [4].

Finally, despite decades of productive research in this emerging field, few transformative advances have been achieved in accessible technologies capable of simultaneously minimizing processing costs and waste generation while improving selectivity and industrial scalability. Significant gaps persist throughout the biomass processing chain, where the specific challenges of each stage are often addressed in isolation rather than as part of an integrated system. To fully realize the industrial potential of microalgae, it is essential to critically identify the bottlenecks inherent to current methodologies and develop scalable technologies capable of effectively overcoming the limitations that still constrain their application.

## 6. Techno-Economic Constraints

The limited scalability of microalgae production remains one of the main obstacles to its consolidation as a competitive food ingredient. Although laboratory and pilot-scale systems often show high productivity, the transition to industrial scale reveals a significant difference between experimental performance and operational viability [7]. From a biological and engineering perspective, scaling up imposes important physical limitations. Although microalgae can theoretically produce 10 to 20 times more protein per hectare than soybeans, this advantage does not always translate into industrial competitiveness due to light attenuation, self-shading, and mass transfer limitations, factors that reduce photosynthetic efficiency and promote heterogeneous growing conditions [102,103,104]. In addition, productivity is highly sensitive to environmental variations, such as temperature, irradiance, and nutrient availability, which can significantly alter biomass composition, especially protein and lipid content [105].

Operational complexity also increases with the scale of production. Open systems have lower implementation costs, but are more susceptible to contamination and crop loss. In contrast, closed photobioreactors offer greater operational control and product quality, but require significantly higher investments in infrastructure and operation [106,107,108].

The economic evaluation of microalgae production requires caution, since the costs reported in the literature vary according to the type of product, degree of processing, level of purity, and final application. The values may refer to whole biomass, protein-enriched ingredients, protein isolates, or purified bioactive compounds, representing different technological, and economic stages. To facilitate this comparison, Table 3 presents representative cost ranges according to the product category and its application.

Under current industrial conditions, whole microalgal biomass remains less economically competitive than conventional protein commodities, despite having high protein content (50–70%). Conversely, higher economic returns are obtained when microalgae are targeted at specialized markets that demand high-purity, functional ingredients or high-value-added bioactive compounds. Even in optimized scenarios, techno-economic analyses estimate production costs between USD 3–6/kg for open systems and over USD 10/kg for closed systems, depending on the technology employed and the location of the production unit [8,109,111]. Thus, achieving competitiveness against conventional protein commodities still requires substantial reductions in production costs.

Energy consumption represents one of the main components of these costs. Operations such as agitation, aeration, artificial lighting, drying, and extraction are highly energy-intensive and can account for 30–40% of the total production cost [112]. This reality contrasts with conventional crops, which depend predominantly on solar energy and require less intensive post-harvest processing [108].

Downstream processing remains another important economic bottleneck. Harvesting and dehydration are particularly challenging due to the low cell concentrations (<1 g/L) and small size of microalgal cells. Although technologies such as centrifugation, filtration, and drying are effective, they are highly energy-intensive and may account for up to 70% of total production costs [113]. Consequently, the harvesting, drying, and extraction steps remain among the main factors limiting the economic viability of large-scale production.

Several strategies have been proposed to improve the technical and economic viability of production, including integration with industrial effluents, utilization of CO_2_ waste streams, and the adoption of biorefinery approaches. Under specific conditions, these strategies can reduce costs to approximately USD 1.5–2/kg of biomass. However, they often depend on highly specialized infrastructures and are not always compatible with regulatory requirements for food. Furthermore, many of these technologies are still at intermediate levels of technological maturity (TRL 4–6), which limits their industrial implementation in the short term [108].

## 7. Industrial Infrastructure, Supply Chains and Market Integration

In addition to the challenges associated with production costs, the expansion of the microalgae sector is limited by structural barriers related to industrial organization, the development of supply chains, and market integration. Unlike conventional agricultural commodities, which have widely integrated and standardized production chains on a global scale, the microalgae industry remains fragmented and decentralized. Production is generally carried out in relatively small units and using different cultivation technologies, which hinders the standardization of processes, the uniformity of product quality, and large-scale industrial integration [114].

The absence of a dominant cultivation technology also delays the consolidation of the sector. Differences between production systems, operating conditions, and processing strategies contribute to high variability in biomass composition and quality, hindering the standardization necessary for large-scale food applications [115,116].

Infrastructure and logistics represent additional challenges. The sector still lacks integrated industrial systems capable of efficiently connecting the stages of cultivation, harvesting, processing, storage, and distribution. Furthermore, the limited stability of fresh biomass often requires immediate processing or controlled storage conditions, reducing logistical flexibility when compared to traditional agricultural commodities [109,111].

The scale of the market is also a significant limitation. While global soybean meal production exceeds 200 million tons per year, global microalgae production remains restricted to only a few tens of thousands of tons annually. Additionally, the different market segments have distinct characteristics: biomass destined for animal feed is associated with high-volume, low-value-added markets, while nutraceutical compounds and functional ingredients serve smaller markets, but with higher economic value [30,110]. This limitation of scale restricts the formation of economies of scale and contributes to maintaining high production costs.

Regulatory fragmentation also represents a significant obstacle to the sector’s expansion. Differences between countries regarding food safety requirements, approval processes, and product classification hinder international trade and increase uncertainty for investors and companies [10]. Furthermore, many of the available techno-economic assessments are still based on pilot systems or simulation models, reflecting the limited industrial maturity of the sector and increasing uncertainty regarding implementation on a commercial scale [112].

More broadly, the current economic model of the microalgae industry remains heavily geared towards the production of high-value-added compounds, such as pigments, DHA and EPA-rich oils, and other bioactive ingredients. Consequently, the economic viability of the sector continues to be sustained mainly by specialized markets, rather than large-scale protein production, highlighting the existing gap between the recognized nutritional potential of microalgae and their current industrial competitiveness [117].

**Figure 2:**
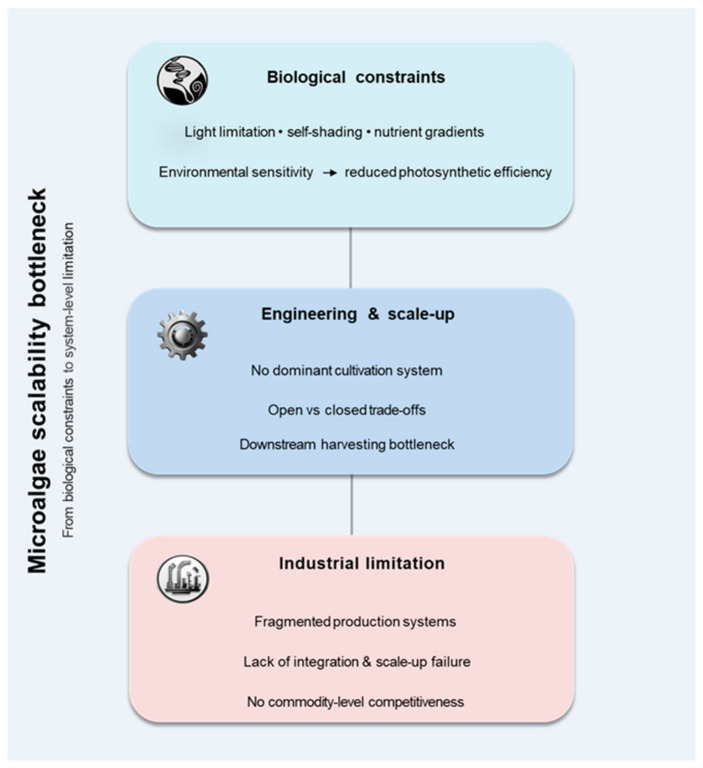
Overview of the main factors limiting the industrial scalability and economic competitiveness of microalgae production. Adapted from: [103,104,118,119,120,121].

In general, the challenges associated with the industrial production of microalgae arise from the interaction of multiple interconnected barriers, rather than a single limiting factor. Figure 2 presents a conceptual overview that relates biological constraints, engineering bottlenecks, and economic outcomes. Overcoming them will require coordinated advances in biotechnology, process engineering, infrastructure development, regulatory harmonization, and market integration. Although progress in improving strains, hybrid cultivation systems, and downstream processing technologies may increase competitiveness, current evidence suggests that microalgae are unlikely to become a dominant source of protein on a large scale in the short term.

## 8. Regulation and Food Safety

In addition to operational limitations, safety regulations often delay the market introduction of microalgae-based products. From a regulatory perspective, microalgae are still considered a relatively novel source of nutrition. Currently, both the United States Food and Drug Administration (FDA) and the European Food Safety Authority (EFSA) stand as some of the most capable organizations regarding the recognition of different microalgae and their commercialization standards. A list of some authorized microalgae by both the FDA and EFSA can be found in Table 4.

Two key legislative frameworks govern the introduction of microalgae-based products into the U.S. market: the Federal Food, Drug, and Cosmetic Act and the Dietary Supplement Health and Education Act. Under the Federal Food, Drug, and Cosmetic Act, any substance intentionally added to food is considered a food additive and, as such, is subject to pre-market evaluation, unless it qualifies for an exemption such as Generally Recognized as Safe (GRAS) status. GRAS labeling is determined on a case-by-case basis through scientific evidence and consensus among qualified experts, and it applies to specific substances under defined conditions of use. As such, different forms of the same microalgal species, such as extracts obtained from whole biomass, may require independent safety assessments [127].

The Dietary Supplement Health and Education Act establishes a separate regulatory pathway for products marketed as dietary supplements. Under this framework, manufacturers are responsible for ensuring the safety of their products and the accuracy of labeling claims. While pre-market approval by the Food and Drug Administration is not required for most dietary supplements, ingredients not previously marketed in the United States before 1994 may be subject to a New Dietary Ingredient (NDI) notification, which must be submitted to the FDA before commercialization [127].

In contrast, the EFSA applies a more precautionary approach, as per the Novel Food Regulation (EU) 2015/2283, if a product was not introduced to the European market with a considerable history of safe use before 15 May 1997, pre-market assessments by the EFSA are required for commercial authorization, this also applies to new products obtained from products that had already been introduced before this date. If an applicant wishes to sell a product they claim to be equivalent to an already authorized product, they may submit a request for notification, whilst providing evidence and research that the two products are equivalent regarding composition, usage, nutritional values and unwanted substances [127].

EFSA safety assessments focus primarily on identification (taxonomic identification and chemical composition) [128], analysis of production processes [129], toxicological analysis on genotoxicity, subchronic toxicity studies and determination of an acceptable daily intake [130], allergenic and nutritional information [129], and a qualified presumption of safety (QPS) based on a list of known microorganisms maintained by the EFSA, that identifies microorganisms presumed to be safe for use in food or feed, bypassing the need for a full assessment for each. If a microalga is missing from this list, a full risk assessment is required.

Both the EFSA and FDA have considerably distinct regulatory practices regarding novel foods, specifically microalgae. While the FDA relies on a decentralized, expert-driven safety determination system, the EFSA enforces a centralized, pre-market authorization process, resulting in a more conservative regulatory landscape for microalgae in the European Union. Regardless of which regulatory practices are put into action, it is fundamental that a legislative body must be well-equipped to work with novel ingredients and their products, especially during an era of change and evolution in the food industry, allowing the general public to have access to healthier alternatives while maintaining the health that has already been achieved at this point.

## 9. Sensory Perceptions and Challenges

Sensory optimization represents a fundamental step toward the successful commercialization of microalgae-based products. Intense coloration, characteristic flavors, and consumer unfamiliarity negatively impact the application of microalgae in food products.

### 9.1. Microalgae Color as a Strength

Color is a major aspect of customer perception and acceptance when deciding on a food product. In this context, the vibrant pigmentation of microalgae can significantly influence consumer perception, serving as either an asset or a liability depending on the product’s positioning and strategic goals [131,132]. In addition, given the universal use of synthetic food dyes, there is significant scope for transitioning toward healthier, large-scale natural alternatives [133,134]. While the chromatic profile of microalgae is frequently viewed as a formulation hurdle or a defect to be suppressed, this perspective overlooks their intrinsic sensory potential and potential uses as food colorants [135]. Thus, this section explores the untapped opportunities within their pigments and organoleptic properties, reframing these remarkable features as strategic strengths.

Essentially, artificial food colorants aim to provide color stability and predictability in the food industry. With standardized food colorants, such as tartrazine (E102), brilliant blue FCF (E133), sunset yellow (INS 110), and ponceau 4R (INS 129), qualitative aspects are more controllable, and shelf life can be extended. Despite their constancy, it is well known that some artificial food colorants may contribute to health issues, such as cancer, allergies, and neurobehavioral changes. Consequently, the application of these food additives is becoming a public health issue [136,137,138]. Moreover, with the rise of the One Health framework on a global scale, customers, companies, and governments are becoming more concerned with the impacts of food production on animal, human, and environmental health. Accordingly, natural food colorants are better for consumers and for the environment [139,140,141,142].

In addition, there is a movement in the research and governmental sectors to create and implement natural alternatives to artificial food dyes [143,144]. In that scenario, microalgae emerge as great potential substitutes for some of the current colorants in the market. They are natural sources of pigments, and within the biomass, there are multiple bioactive compounds with positive effects on human health. However, their natural pigments are sensible under high intensity of light, heat or pH changes, what makes it more difficult and expensive to apply them on a wide range of industrial processes [145,146].

Due to microalgae bright colors, they emerge as candidates for multiple natural dye sources. Thus, some frameworks are possible. In a low-complexity approach, the microalgae biomass can be dried and utilized straightaway in food production. It will result in products with the color tones of the chosen microalgae. Additionally, the biomass can affect the physical and chemical properties of the formulations [147,148]. An aspect that provides flexibility with this straightforward solution is natural variety. Commercial microalgae strains encompass a variety of biomass colors, such as green, yellow, red, blue, and golden shades, serving as a comprehensive source of natural colorants intended for industrial use [149,150,151,152,153,154].

Following this straightforward approach, spray-dried microalgae was applied as a natural food dye in ice cream, achieving a variety of color tones. However, while the coloring was satisfactory, the primary concern remained the characteristic odor and taste of the microalgae, which negatively impacted the sensory results [155]. Another tested application is the use of *A. platensis* BEA 005B biomass for the replacement of an artificial green colorant. During the baking procedure of the macarons, the achieved temperatures were moderately high, around 121 °C for 15 min, which helped to reduce the degradation of the color when compared to other baking applications. However, color changes continued to occur during the storage period, in various temperatures (15 °C, 30 °C, 60 °C) and still, the strong coloring power of the biomass was confirmed. The quantity applied did not increase the bioactive capacity of the macarons, when compared to the control [156]. Also, Spirulina powder was validated for beer production, partially replacing malt in 5%. The use of Spirulina powder in the beers did not affect the fermentation processes. Panelists preferred the beers prepared with an India pale ale (IPA) method, which achieved a strong blue color and an acceptable microalgal taste [157].

Remarkably, the diversity of carotenoids and chlorophylls present in microalgal cells allows a wide range of possible applications. Therefore, the prepared food product will incorporate not only all the nutrients in the biomass and its color, but also the taste and odor of the biomass, and this could represent a challenge, depending on the necessary biomass amount. Nevertheless, utilizing microalgal biomass as a natural food colorant presents certain constraints. As mentioned in several studies, microalgae are often related to fishy and earthy notes, which can be undesired in some food matrices. Although the use of whole microalgal biomass from different strains is relatively cost-effective and adaptable, appropriate formulation strategies and sensory optimization can reduce the need for extracting specific pigments. Particular attention should be given to pH, thermal processing conditions, and interactions with other formulation ingredients, as these factors play a critical role in improving color stability and consumer acceptance [158,159,160].

A further feasible strategy is the industrial extraction of concentrated natural pigments derived from microalgal biomass. For example, although Spirulina biomass is naturally green, it serves as a source for green, yellow-to-orange, and blue pigments. The ability to derive multiple colorants from a single source offers substantial strategic flexibility for the food industry. Additionally, the richness of microalgal biomass is a key asset for bio-refinery setups, with a circular economy organization. In this kind of industry, microalgae can serve as a source of natural food dyes, proteins, and lipids, for example, reducing waste and pollution. Naturally, each microalgal strain will present a unique profile and distribution of bioactive compounds inside the cells [161,162,163,164,165].

**Figure 3:**
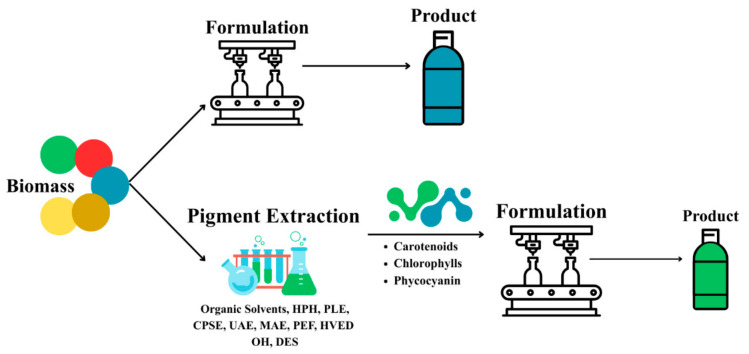
Comparison of microalgal biomass processing routes for product development. The diagram illustrates two pathways: the direct use of biomass in product formulation (**top**) and the specialized Pigment Extraction route (**bottom**). While direct use is simpler, the extraction of specific compounds like carotenoids, chlorophylls, and phycocyanin, utilizing methods such as organic solvents, pressurized systems (HPH, PLE, CPSE), wave-energy (UAE, MAE), electrical treatments (PEF, HVED, OH), or green solvents (DES), allows for enhanced product stability, extended shelf-life, and the removal of undesired flavors, despite requiring higher initial investment in equipment and energy.

In this manner, the process to extract single pigments from microalgal biomass can represent a larger investment in equipment and energy (Figure 3). Simultaneously, large-scale production can lead to enhanced stability, extended shelf-life, and the mitigation of undesired off-flavors [166,167,168]. Currently, there are some available methods to isolate interest pigments from microalgal biomass. Traditional approaches include the use of solvents to extract the pigments from cells. Organic solvents are quite efficient in this task; there is a concern of toxicity for food applications [169]. On the search for safer extraction protocols, other solutions have been emerging, such as pressurized systems, represented by high-pressure homogenization (HPH), pressurized liquid extraction (PLE), and continuous pressurized solvent extraction (CPSE). Pressurized systems apply a combination of mechanical impacts, turbulence, cavitation, or controlled high pressures to break cells. Additionally, there are systems utilizing Wave-energy treatments, such as, for example, ultrasound-assisted extraction (UAE) and microwave-assisted extraction (MAE); these approaches are efficient in lowering processing times and temperatures, a concern in large-scale applications. Moreover, other solutions include enzymatic extraction, pulsed electric fields (PEF), high voltage electrical discharge (HVED), and ohmic heating (OH) [170,171,172,173,174,175,176,177].

From a greener standpoint, Deep Eutectic Solvents (DES) represent a sustainable extraction framework. Composed of renewable compounds such as quaternary ammonium salts, they are non-toxic to ecosystems and human health. Furthermore, their relatively low production cost enhances their industrial viability [178]. DES is a promising solution to integrate circular biorefinery approaches, where many interesting compounds can be extracted from the same biomass. Nevertheless, the use of DES itself does not guarantee efficiency and reliability of the extraction; the protocol needs to be adapted to each industrial setup [179,180,181]. For instance, to extract astaxanthin from *Haematococcus pluvialis*, a two-phase system based on deep eutectic solvent followed by liquid–liquid extraction was able to achieve a 99% efficiency, which is an optimal condition [182], and not all large-scale extractions will achieve such high efficiency [183].

### 9.2. Strategies for Flavor Enhancing

Remarkably, the fishy, earthy, and distinctive odorant characteristics of microalgae do not originate from a single compound [184]. The volatile compound breakdown can vary from species to species and with more advanced methodologies, even new compounds are being unraveled as responsible for the microalgal sensory profiles perceived by humans. For instance, in 2025, with an offline and online fractionation approach, 14 new compounds were discovered in commercial Spirulina biomass and documented for the first time. This analysis found compounds with flavor dilution (FD) factors between 8 and 2048. Among the found constituents, the most potent substances identified included crustaceous-like, earthy, 2-ethyl-3,5-dimethylpyrazine (FD 2048), vinegar-like acetic acid (FD 1024), floral, violet-like β-ionone (FD 1024), and sweaty 2- and 3-methylbutanoic acid (FD 2048). Moreover, the study also identified a sulfuric, cabbage-like methanethiol (FD factor ≥ 32) [185].

Curiously, the molecule 2-ethyl-3,5-dimethylpyrazine can be found in other natural products like sunflower seeds, *Bacillus subtilis*, the red imported fire ant (*Solenopsis invicta*), peanut, hazelnut, pumpkin, and black pepper oil. It is associated with a strong roasted aroma and has a low odor threshold, being easily perceived, even in small concentrations. There is an interest in the potential use of this isolated compound as a food odorant in the industry [186,187,188,189].

During cultivation, it is possible to change the environment where the microalgae grow in order to alter their organoleptic and biochemical qualities. For instance, by adding a supply of nitrogen in the medium of *Tetraselmis chuii*, it is possible to decrease earthy notes, increase umami, and salty taste [190]. Moreover, by employing a co-cultivation method with *Saccharomyces cerevisiae* and *A. platensis* (yeast/microalgae biomass ratio of 10:1000), the microalgae reached a biomass with a light green color and “floral–fruity” aromas, and no volatile sulfur compounds [191]. Though if the harvesting process was not enough to achieve the desired organoleptic properties, some processing options may be available for the biomass [192].

With the growing interest in microalgae as novel ingredients in the food sector, new strategies for flavor enhancement and neutralization have been emerging. For instance, Chlorella vulgaris, a widely available commercial microalgae, has remarkable leafy aromas, mostly brought by hexanal aldehydes. A fermentation technique utilizing lactic acid bacteria (LAB) strains is very effective in decreasing the concentration of these aromatic compounds. The best results in this assay were attributed to the LAB Lacticaseibacillus paracasei UPCCO 2333, which was able to decrease the amount of ketones, aldehydes, pyrazines, and terpenes, even though it enhanced the ester production [193]. Other strains of Lacticaseibacillus paracasei have been present in large-scale productions in the food industry for decades. This kind of fermentation is easy to manage on an extensive scale, inexpensive to maintain, and safe for human nutrition purposes. The use of LAB could overcome many challenges in the microalgae implementation [194,195].

Therefore, fermentation emerges as a low-cost, effective, and safe technique to enhance flavors and aromas in microalgae. Fermentation tends to increase the appearance of floral, fruity, and buttery traces, due to the production of esters, higher alcohols, and a variety of other aromatic molecules. It decreases tetramethyl pyrazine (responsible for roasted, nutty notes) and hexanal aldehyde (green, leafy aromas). Also, some interesting flavor compounds are found in fermented microalgae, such as isoamyl alcohol (related to banana and whisky) and 3-methyl butanoic acid (dairy notes and acidic). Moreover, fermented biomass presents increased aspartic and glutamic acids, leading to an improved taste, enhancing capability, and masking of undesired notes [196,197].

Yet fermentation is not the only solution to improve the acceptability of microalgae. Similarly, León-Door et al. [198] compared three different methods for deodorization of *A. platensis* before applying the biomass in yogurt enrichment. Adsorption by activated carbon, extraction with ethanol, and fermentation with *Saccharomyces cerevisiae* were evaluated. Yogurts with fermented microalgae showed the greatest sensory scores (>8.7/10). Products that contained untreated biomass or activated carbon treatment had lower scores, with acceptability at small enrichment proportions (≤0.5%). On the other hand, ethanol treatment successfully decreased aldehydes and ketones (Safranal and β-Ionone), though fermentation eradicated pyrazines and produced acceptable alcohols and acids (1-Pentanol and Butanoic acid).

Therefore, strategies to enhance the taste, odor, and color of microalgae-based applications are in constant development. These new approaches are designed in alignment with consumer research to understand how different segments interact with such products, as well as to capture their insights and future expectations. Combining updated data on consumer perception with innovative industrial applications is essential to keep pace with evolving consumer behavior [199,200,201].

### 9.3. Neophobia and Consumer Attitudes Towards Microalgae

Despite its apparent simplicity, the process of food selection and consumption is far from trivial, encompassing a complex array of perceptions, preferences, and decision-making mechanisms. Such decisions occur within milliseconds and are strongly influenced by biological, physiological, psychological, and environmental factors [202]. Moreover, decisions regarding food acquisition and preparation are inherently linked to cultural norms and the consumer’s environment, including factors such as upbringing, geographical location, age, and beliefs [203,204,205,206]. Consequently, applied methodologies such as ethnography are being combined with Food Choice Questionnaires (FCQ) to gain a deeper understanding of consumer behavior and perceptions from multiple perspectives [207,208,209].

In this scenario, a major issue regarding the acceptance of microalgae in contemporary diets is food neophobia. This is characterized as the fear or resistance to experiencing new products outside the consumer’s regular diet. Neophobia levels can fluctuate throughout life and are impacted by environmental and lifestyle changes [210]. A food neophobia scale was first developed by Pliner and Hobden in 1992 [211]. In the past decades, multiple alternative scales have been created in order to adapt the methodology to contemporary times and overcome present challenges [212,213,214,215]. It is reported that individuals with lower levels of neophobia are more open to including microalgae in their daily routines. In addition, increasing the awareness of the aspects surrounding microalgae, such as sustainability and health benefits, can decrease feelings of uncertainty and increase confidence in novel food items [199,216,217].

Notably, consumer attitudes toward microalgae vary according to country, demographic segmentation, and geographical location. Several studies have mapped these perceptions across consumers from diverse backgrounds. For instance, research involving Singaporean consumers demonstrated that tofu and seaweed were perceived as more natural, festive, and sustainable than microalgae. Furthermore, within the Singaporean market, microalgae-based meat and fish substitutes emerged as the most appealing options for the adoption of microalgal protein [218]. Looking at the European market, the scenario presents additional challenges. First, there are significant regulatory hurdles in Europe, as microalgae are not traditionally consumed in the region. This is reflected in European consumer behavior: while microalgae are considered an acceptable food additive, high levels of incorporation are often rejected due to undesirable color changes and off-flavors. Furthermore, freshwater microalgae are generally preferred over marine species, primarily due to the absence of fishy sensory notes [219,220,221].

A recent consumer attitude survey conducted in the Netherlands, Germany, Hungary, Spain, and Italy revealed similar trends regarding the perception of microalgae-based foods. Microalgae remain relatively unfamiliar to many consumers, with nearly half of respondents reporting no prior exposure to microalgae during their lifetime. Overall, willingness to try microalgae-based products and confidence in their potential benefits were moderate. The study identified distinct consumer segments, including enthusiasts, cautiously curious individuals, undecided consumers, and those uninterested in microalgae-based foods. Consumers who were more willing to experiment with such products generally associated microalgae with innovation, naturalness, and premium pricing. Moreover, individuals aware of the nutritional potential and production costs of microalgae tended to expect clean-label products offering substantial health benefits. Environmental awareness also played a positive role, as consumers concerned about climate change were more receptive to the incorporation of microalgae into their diets. Similarly, adherence to flexitarian dietary patterns was associated with a greater willingness to try microalgae-based foods [222].

**Figure 4:**
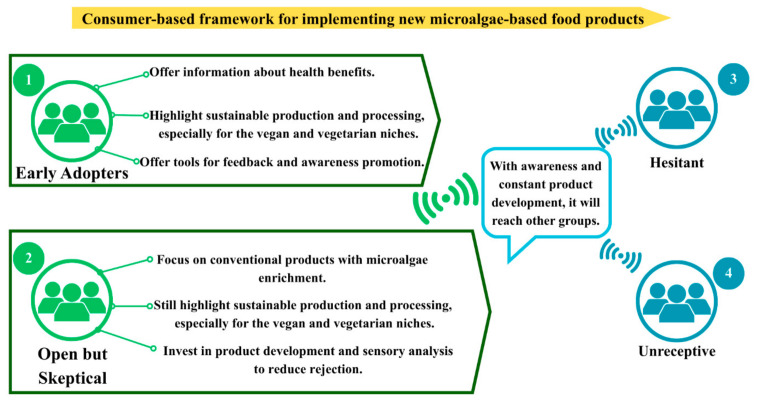
Consumer-based framework for implementing new microalgae-based food products. The diagram outlines strategic approaches tailored to distinct consumer segments: prioritizing health benefits and sustainability messaging for Early Adopters (Group 1), and focusing on sensory optimization and enrichment of conventional products for the Open but Skeptical (Group 2). Through targeted awareness and iterative product development, these strategies aim to generate a “ripple effect” to eventually engage Hesitant (Group 3) and Unreceptive (Group 4) demographics, effectively overcoming barriers such as flavor rejection and lack of familiarity.

Moreover, in Brazil, a trial with 1499 volunteers found that 51% of them had already heard about microalgae. Nevertheless, 72% never tried any foods with them, but 84% would be open to tasting this kind of product. Individuals showcased a preference for the application of microalgal products in food supplements (73%), seasonings (56%), baked goods (49%), sauces (44%), drinks (32%), and dairy (2%). Almost half of the volunteers stated that they would be willing to pay 5% to 10% more for microalgal products [223]. Based on these combined perspectives, there is the possibility of a customer-oriented novel strategic framework for implementing microalgae-based food products in the market (Figure 4). This new strategic framework targets early adopters to gain initial market share and refine product quality through their feedback. Consequently, a more robust product and marketing strategy will gradually reach risk-averse individuals, maximizing the chances of acceptance and long-term engagement [224,225].

Likewise, an examination conducted with Swiss consumers elucidated some key points about their inclinations. The individuals who were more open to consuming microalgal products presented less neophobia, a concern with the environmental impact of their diets, and a previous negative view of the sustainability of the meat industry. They trust that products from microalgae will be better for the planet when compared to animal protein. In addition, they related that they prefer microalgae products that mimic meat sensory properties [122]. In Austria, researchers utilized crackers to investigate the customer attitude, with a discrete choice experiment (DCE). The willingness to pay was higher for organic microalgae-based items, especially when compared to similar studies, and price was very relevant for the decision [226]. In addition, consumers do not perceive microalgae as regular ingredients or common food products. They are inclined to associate them with superfoods or supplements, more connected to pharmaceutical products than something they will look for on supermarket shelves. Of course, the relevance of microalgae for the pharmaceutical industry is vast; however, when targeting food consumers, strategies need to be adapted, in order to educate consumers about this new application [227].

On a similar line, a study took place in Indonesia, a tropical country with great potential for microalgae production. In this occasion, a few consumer groups were identified. On this framework, the “Independent Knowledge Holders” are solely based on their own knowledge about sustainable foods, while the “Curious but Restricted” need a little push to take an attitude, driven by curiosity. On the opposite side, the “Informed Passive Receivers” are not resistant to new food experiences but do not actively explore the market. Lastly, “Sustainability-Driven Neophobes” are very open and active in searching for new sustainable products [228]. These findings open the discussion to new strategies for the implementation of microalgal food products. Identifying consumer groups helps to adequate initiatives to a smaller public and utilize their influence to impact a larger group. For instance, marketing strategies can be adopted to drive curiosity in a specific group, as they perceive a new food coming into the market that is already consumed and trusted by a more active consumer group [224].

Remarkably, Migliore et al. [229] conducted a systematic scoping review inspecting aspects influencing consumer attitudes, intentions and reception of *A. platensis*. The review highlights a gap in market studies in different regions of the globe, different countries, continents, and cultures. Most of the consumer research was conducted in Europe, with a few in Asia and other locations. This systematic review emphasizes the positive influences and boundaries identified by targeted research. Health and nutrition benefits, sustainability, interest in innovation, and social trends appear as highly relevant drives when persuading consumers. On the other hand, there is a concern about sensory experience, unsustainable production methods, high price, and lack of familiarity with their usual diets. Zhou et al. [174] suggest that new advertising strategies can contribute to overcoming the initial fear barrier. As discussed by [179], adding microalgae to a traditional food may be achievable if the consumers are advised and educated on the benefits and advantages of the new formulation. Younger individuals, with better access to information and health-oriented products, may present a better acceptance rate, if microalgae are inserted in conventional products [230].

Another feasible approach to overcome neophobia and mask unexpected sensory properties in food consists of inserting microalgae into products with similar expected characteristics. Thus, microalgal biomass possesses the ideal functional properties for some specific food applications, such as seafood analogues, still a limitedly explored commercial field [222,231,232]. For instance, the presence of alanine, glutamic acid, arginine, and 5′-ribonucleotides present in microalgae contributes to the seafood taste perception in consumers [197,231]. Combined with other pigments, fat, fillers, and additives, multiple methods can be used to create vegan seafood analogues. Extrusion, wet spinning, electrospinning, and 3D printing are some of the possibilities for the industry to explore while developing new desirable products [233]. Seafood plant-based alternatives can be produced with a range of vegan proteins (soy, pea, rice), and microalgae can be adopted as the physical base or an additive for improving structure, odor, and flavor [230,231,232,233,234].

## 10. Conclusions and Future Perspectives

Finally, microalgae represent a highly promising platform for the development of sustainable and functional food ingredients due to their remarkable nutritional composition, bioactive potential, and technofunctional properties. Despite this, the large-scale integration of microalgae into the food industry remains limited by technological, economic, sensory, and regulatory barriers.

High production costs, limited industrial scalability, biomass variability, energy-intensive processing, and consumer resistance associated with characteristic flavors and colors continue to restrict wider market adoption. In addition, regulatory complexity and a lack of standardized production systems further delay industrial consolidation. Although several emerging technologies have demonstrated potential to improve biomass processing, extraction efficiency, and sensory quality, many approaches remain limited to laboratory or pilot scales.

Future advances in the sector will depend on multidisciplinary strategies that integrate biotechnology, process engineering, food science, and market research. In particular, the development of scalable cultivation systems, low-cost downstream processing technologies, ecological extraction approaches, and sensory optimization strategies will be essential to improve industrial viability. In addition, biorefinery concepts and circular economy approaches can contribute to increasing the sustainability and economic competitiveness of microalgae production.

Overall, while microalgae are unlikely to replace conventional food systems in the short term, they represent a strategic resource for the development of next-generation functional foods and sustainable ingredients. Their effective consolidation in the global food market will require coordinated scientific, technological, industrial, and regulatory advances capable of bridging the gap between experimental potential and large-scale commercial application.

## Figures and Tables

**Table 1:** Nutritional and caloric value of commercial microalgae and conventional food sources.

Microalgae/Food Matrices	Caloric Value (kcal/100 g) ^a^	Content (g/100 g) ^ab^				Dry Weight Content (%) ^bcd^			
		Protein	Carbohydrate	Lipid	Ash	Protein	Carbohydrate	Lipid	Ash
*A. platensis* (Spirulina)	290	56	24	8	6	60–70%	15–20%	5–8%	7%
*Chlorella vulgaris*	300–400	60	20–40	0–10	nf ^e^	42–60%	12–55%	5–40%	5%
*Dunaliella salina*	nf ^e^	nf ^e^	nf ^e^	nf ^e^	nf ^e^	19–57%	6–40%	18–43%	7–9% ^bd^
*Haematococcus pluvialis*	nf ^e^	nf ^e^	nf ^e^	nf ^e^	nf ^e^	29–45%	15–63%	20–25%	1.6% ^f^
Beef	260	19	0	15–20	0.9	56–79%	0%	21–45%	0–2% ^g^
Chicken breast	106–119	21–23	0	3	1	88–90%	0%	9–12%	1–2% ^h^
Egg	146–150	12	0.6–0.9	10	1	57%	0.6–3%	41–57%	0.5% ^i^
Potato	52–77	1–2	12–16	0–3	0.9	9%	91%	0%	4–5% ^j^
Rice	129	3	28	0.3	nf ^e^	8%	91%	0.6%	0.3% ^k^

^a^ [14]; ^b^ [15]; ^c^ [16]; ^d^ [17]; ^e^ Not found in the USDA and TACO database; ^f^ [18]; ^g^ [19]; ^h^ [20]; ^i^ [21]; ^j^ [22]; ^k^ [23].

**Table 2:** Techno-functional properties presented by microalgae.

Techno-Functional Properties	Compounds	Mode of Action
Oil/water absorption, emulsification, foaming	*A. platensis*	Techno-functional enhancement with pH-boosted oil absorption even with low protein concentration. Foaming properties similar to egg proteins.
Gelation	*C. sorokiniana*	They form gels when heated.
Emulsifier solubility	*C. vulgaris*	Enhanced emulsifying properties of proteins. Solubilization of the extracted protein.
Water absorption	*D. salina*	Rheological properties of incorporated microalgae.
Emulsifier	*H. pluvialis*	Strong emulsifying potential of isolated proteins.
Solubility	*Nannochloropsis oculata*	Maximum solubility between pH 7 and 10.
Solubility	*Tetraselmis* sp.	Solubility is independent of ionic strength and appropriate pH.
Foam formation	*Tetraselmis* sp.	Formation of stable foams through selective adsorption at the air-water interface.
Gelation	*Tetraselmis* sp.	Superior surface activity and gel formation compared to whey protein isolate.
Solubility, foam stability	*Chlamydomonas reinhardtii*	etter foam stability compared to *S. platensis* and *S. maxima*.
Water/oil binding, emulsification, foaming capacity	*Euglena gracilis*	Greater water/oil binding capacity, emulsification, and stable foaming due to high surface hydrophobicity and β-sheet content.

Adapted from [30,64].

**Table 3:** Economic comparison by product category.

Product Category	Typical Product	Approx. Cost Range	Protein Content/Purity	Main Application
Bulk agricultural commodity	Soybean meal	USD 1–2/kg	~40–50% protein	Food/feed commodity
Dairy protein ingredient	Whey protein concentrate	USD 5–10/kg	~35–80% protein	Food formulation/supplements
Feed-grade protein source	Plant/soy feed meal	USD 1–10/kg	Variable	Animal nutrition
Whole microalgal biomass	*Spirulina*, *Chlorella*	USD 10–50/kg	~50–70% protein	Food/feed/functional ingredient
Microalgae protein isolate	Purified microalgal protein	USD 30–70/kg	>70–90% purity	Functional food ingredient
High-value bioactive compounds	Astaxanthin, DHA/EPA, pigments	USD 100–300/kg compound	Compound-specific purity	Nutraceutical/pharma

Adapted from [8,30,109,110,111,112].

**Table 4:** Microalgae authorized by the EFSA and GRAS-labeled by the FDA.

Microalga	EFSA Status	FDA Status	References
*A. platensis*/*maxima* (Spirulina)	Non-novel (consumed pre-1997)	GRAS	[122,123]
*Chlorella vulgaris*/*pyrenoidosa*	Non-novel (consumed pre-1997)	GRAS	[123]
*Haematococcus pluvialis*	Authorized Novel Food (*astaxanthin*)	GRAS	[123,124]
*Schizochytrium* sp.	Authorized Novel Food (DHA oils)	GRAS	[123,125]
*Ulkenia* sp.	Authorized Novel Food	GRAS	[123]
*Tetraselmis chuii*	Authorized Novel Food + QPS	GRAS	[7,126]
*Dunaliella salina*	Authorized (β-carotene aditive)	GRAS	[123]

## Data Availability

No new data were created or analyzed in this study. Data sharing is not applicable to this article.
